# Influence of menopause on chemotherapy‐induced nausea and vomiting in highly emetogenic chemotherapy for breast cancer: A retrospective observational study

**DOI:** 10.1002/cam4.6494

**Published:** 2023-09-07

**Authors:** Takashi Yokokawa, Kenichi Suzuki, Daiki Tsuji, Mari Hosonaga, Kazuo Kobayashi, Kazuyoshi Kawakami, Hitoshi Kawazoe, Tomonori Nakamura, Wataru Suzuki, Takahito Sugisaki, Takeshi Aoyama, Koki Hashimoto, Masahiro Hatori, Takuya Tomomatsu, Ayaka Inoue, Keiichi Azuma, Maimi Asano, Toshimi Takano, Shinji Ohno, Masakazu Yamaguchi

**Affiliations:** ^1^ Department of Pharmacy Cancer Institute Hospital of Japanese Foundation for Cancer Research Tokyo Japan; ^2^ Department of Clinical Pharmacology, School of Pharmacy Tokyo University of Pharmacy and Life Sciences Tokyo Japan; ^3^ Department of Clinical Pharmacology and Genetics, School of Pharmaceutical Sciences University of Shizuoka Shizuoka Japan; ^4^ Breast Oncology Center Cancer Institute Hospital of Japanese Foundation for Cancer Research Tokyo Japan; ^5^ Division of Pharmaceutical Care Sciences Keio University Graduate School of Pharmaceutical Sciences Tokyo Japan

**Keywords:** breast cancer, chemotherapy‐induced nausea and vomiting, estradiol, follicle‐stimulating hormone, menopause, risk factor

## Abstract

**Background:**

Female sex and younger age are reported risk factors for chemotherapy‐induced nausea and vomiting (CINV) in highly emetogenic chemotherapy, but the underlying mechanism has not been elucidated. The purpose of this study was to clarify the impact of menopause on CINV.

**Methods:**

This retrospective observational study analyzed data from consecutive patients who received their first cycle of perioperative anthracycline‐based chemotherapy for breast cancer between January 2018 and June 2020. The endpoints were association between CINV (vomiting, ≥Grade 2 nausea, complete response [CR] failure) and menopause as well as the association between CINV and follicle‐stimulating hormone [FSH]/estradiol [E2].

**Results:**

Data for 639 patients were analyzed. Among these patients, 109 (17.1%) received olanzapine (four antiemetic combinations) and 530 (82.9%) did not (three antiemetic combinations). Premenopausal state (amenorrhea lasting ≥12 months) was significantly associated with ≥Grade 2 nausea and CR failure in univariate analysis but not in multivariate analysis. The premenopausal FSH/E2 group (defined by serum levels; FSH <40 mIU/mL and E2 ≥20 pg/mL) had a significantly higher rate of ≥Grade 2 nausea than the postmenopausal FSH/E2 group (FSH ≥40 mIU/mL and E2 <20 pg/mL) (48.8% vs. 18.8%, *p* = 0.023).

**Conclusions:**

Our results suggest that changes in FSH and E2 due to menopause may affect the severity of nausea and that FSH and E2 (especially FSH) levels might be useful indicators for CINV risk assessment.

## 1 INTRODUCTION

Anthracycline‐based chemotherapy is categorized as a highly emetogenic chemotherapy (HEC) according to multiple antiemetic guidelines.^1^, ^2^, ^3^, ^4^ For perioperative AC (doxorubicin and cyclophosphamide) and FEC100 (fluorouracil, epirubicin, and cyclophosphamide) therapy in breast cancer, maintenance of relative dose intensity (RDI) contributes to patients' survival.^5^, ^6^ On the other hand, chemotherapy‐induced nausea and vomiting (CINV) attenuates the RDI.^7^ Therefore, control of CINV is extremely important.

The reported complete response (CR; no vomiting/retching and no rescue medication) rate during the 0–120 h period in HEC is 34%–55% for the three antiemetic combinations (neurokinin‐1 receptor antagonist [NK_1_ RA], 5‐hydroxytryptamine type‐3 receptor antagonist [5‐HT_3_ RA], and dexamethasone)^8^, ^9^, ^10^ and 50%–64% for the four antiemetic combinations (NK_1_ RA, 5‐HT_3_ RA, dexamethasone, and olanzapine).^9^, ^10^ However, as there are individual differences in the degree of symptoms, it is necessary to select an appropriate antiemetic therapy that takes this into consideration. As previous studies have reported, female sex, younger age, no drinking habits, and history of morning sickness are patient‐related risk factors for nausea and vomiting.^11^, ^12^, ^13^ A systematic review of pharmacogenomics studies showed that *ABCB1* polymorphisms represent a genetic factor affecting antiemetic efficacy.^14^ Multiple studies have shown that female sex and younger age in particular are established risk factors,^11^, ^12^, ^13^, ^15^, ^16^, ^17^, ^18^ but the underlying biological mechanism remains unknown.

A previous study reported that early postoperative nausea and vomiting (PONV) is more frequent in premenopausal women, who also require more rescue antiemetic medication.^19^ However, no reports have shown an association with menopause in CINV. In contrast, several reports have shown an association between CINV and history of morning sickness.^12^, ^13^ An association between morning sickness and hormone secretion has also been reported, and pregnant women with high human chorionic gonadotropin (hCG) levels reportedly experience more symptoms of morning sickness.^20^ hCG increases progesterone and estrogen secretion.

Based on the above observations, we hypothesized that menopause and female hormones play a role in the mechanism of CINV. However, there are no reports of studies examining this relationship. The purpose of this study, therefore, was to clarify the effects of menopause and female hormones on CINV.

## 2 MATERIALS AND METHODS

### 2.1 Study design

This was a retrospective cohort study using data from a single center. Eligibility criteria included consecutive patients who received perioperative anthracycline‐based chemotherapy for early‐stage breast cancer at the Cancer Institute Hospital of the Japanese Foundation for Cancer Research between January 2018 and June 2020. Exclusion criteria were as follows: (i) male sex, (ii) patients with a history of chemotherapy, (iii) dose reduction of anticancer agents in the first cycle, (iv) administration of non‐standard antiemetic treatment, (v) administration of concomitant drugs having a preventative effect against nausea and/or vomiting, and (vi) patients with missing data.

### 2.2 Treatment regimen and study drugs

All patients received anthracycline‐based chemotherapy via intravenous infusion every 3 weeks (AC regimen: doxorubicin [60 mg/m^2^] and cyclophosphamide [600 mg/m^2^] or FEC100 regimen: epirubicin [100 mg/m^2^], cyclophosphamide [500 mg/m^2^], and 5‐fluorouracil [500 mg/m^2^]).

As supportive care drugs, patients were given three antiemetic combinations (oral aprepitant [125 mg on Day 1 and 80 mg on Days 2 and 3], intravenous palonosetron hydrochloride [0.75 mg on Day 1], and dexamethasone‐phosphate sodium [dexamethasone; 12 mg {equivalent to 9.9 mg of dexamethasone} on Day 1 and oral dexamethasone {8 mg on Days 2–4}]) or four antiemetic combinations (oral olanzapine [2.5–5 mg] on Days 1–4 [maximum: up to Days 6], in addition to the abovementioned three‐drug combination of aprepitant, palonosetron, and dexamethasone).

### 2.3 Endpoints

Endpoints were as follows: (i) association between CINV and menopause, and (ii) association between CINV and female hormones, specifically follicle‐stimulating hormone (FSH) and estradiol (E2), which are used in the criteria for menopause. Nausea and vomiting were graded according to Common Terminology Criteria for Adverse Events, version 5.0. CR failure was defined as failure to achieve CR, that is, patients with vomiting/retching episodes and use of rescue antiemetic medication. The observation period was set as the first cycle.

### 2.4 Statistical analysis

Univariable and multivariable logistic regression analyses according to the forced entry method were used to evaluate any association between CINV and menopause. To account for indication bias due to lack of randomization, we performed multivariable analyses consisting of the following explanatory variables as covariates. The objective variables were analyzed as vomiting (all grades), ≥Grade 2 nausea, and CR failure. The explanatory variables included age, body mass index, alcohol consumption habits, history of morning sickness, chemotherapy regimen, antiemetic regimen selected from previous studies as patient‐related risk factors,^11^, ^12^, ^13^, ^15^, ^16^, ^17^, ^18^, ^21^ and menopause (physician's diagnosis: amenorrhea lasting ≥12 months) selected from a clinical perspective as an unknown factor. Note that although AC and FEC100 are both anthracycline‐based regimens, they have different CINV risks and have been reported to have divergent CR rates, so we included chemotherapy regimen as one of the explanatory variables.^8^ Before performing the multivariate logistic regression analysis, the variance inflation factor (VIF) was calculated to determine if there was multicollinearity between the explanatory variables. If multicollinearity (VIF ≥10) was observed, multivariate logistic regression analysis was performed using only one explanatory variable. Regarding associations between CINV and FSH/E2, the serum levels of FSH and E2 quantified by electrochemiluminescence immunoassay before the start of chemotherapy were divided into two groups based on the criteria^22^ for menopause. CINV rates were then compared between the premenopausal FSH/E2 group (i.e., FSH <40 mIU/mL and E2 ≥20 pg/mL)^22^ and the postmenopausal FSH/E2 group (i.e., FSH ≥40 mIU/mL and E2 < 20 pg/mL)^22^ using the chi‐squared test or Fisher's exact test. In addition, the analysis was performed in terms of FSH and E2, respectively. CINV rates were then compared between the Low FSH group (<40 mIU/mL) and High FSH group (≥40 mIU/mL) and the Low E2 group (<20 pg/mL) and High E2 group (≥20 pg/mL) using the chi‐squared test or Fisher's exact test. All significance levels were set to 5%. For statistical analysis, SPSS software, version 24.0 (SPSS), was used.

The sample size necessary to verify the purpose of this study was calculated beforehand using the Monte Carlo method.^23^ On the basis of previous studies,^21^, ^24^ we assumed that the incidence of vomiting would be 11%. We estimated that we would need to enroll approximately 637 patients to evaluate the seven input explanatory variables by multivariate logistic regression analysis.

### 2.5 Data collection

Data were obtained from electronic medical records. The study protocol was approved by the Clinical Research Ethics Review Committee of the Cancer Institute Hospital (approval number: 2019‐GA‐1238) and conducted in accordance with the Declaration of Helsinki.

## 3 RESULTS

### 3.1 Patient characteristics

**FIGURE 1:**
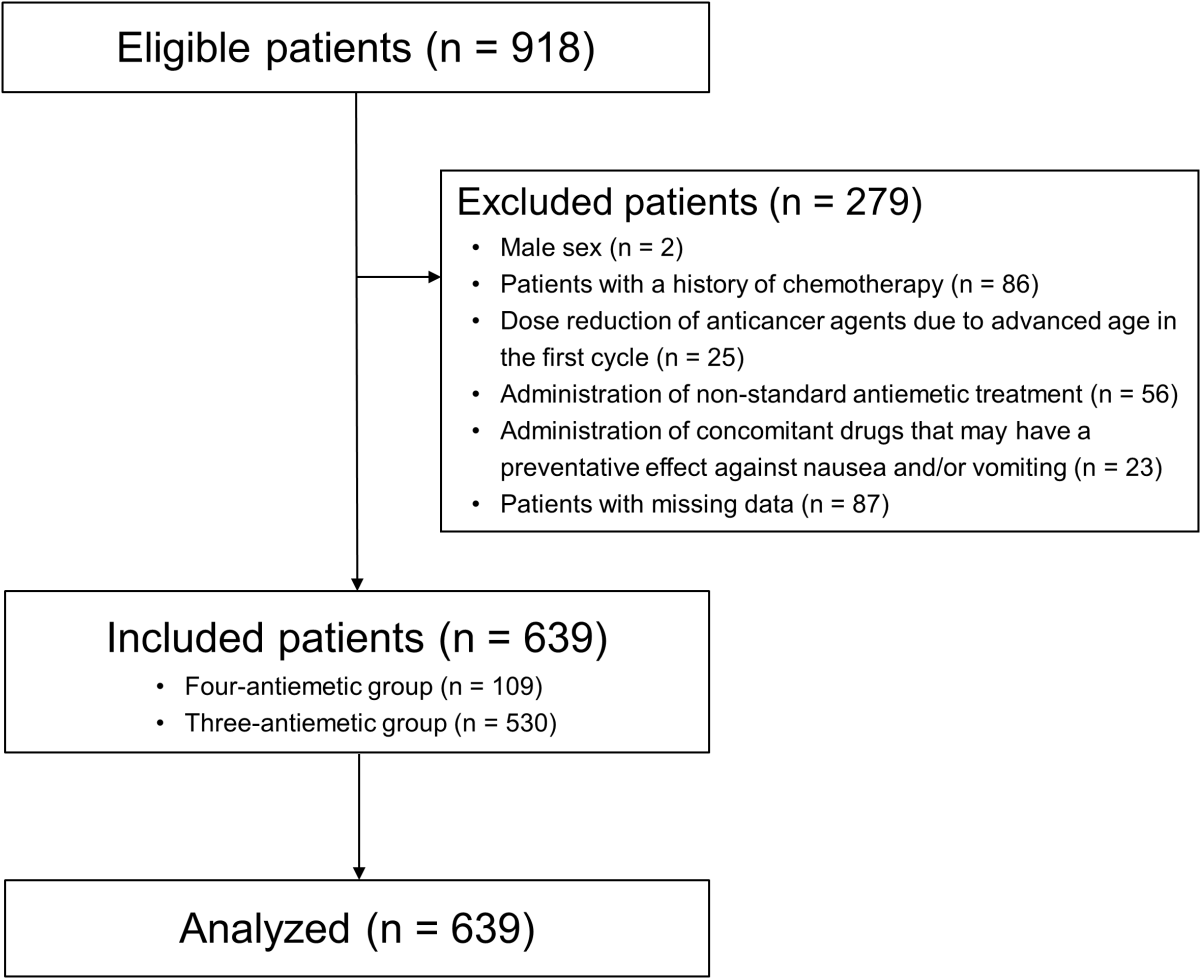
Patient enrollment flowchart. Four‐antiemetic group: Palonosetron + Aprepitant + Dexamethasone + Olanzapine; Three‐antiemetic group: Palonosetron + Aprepitant + Dexamethasone.

A flowchart describing patient enrollment is shown in Figure 1. Of the 918 patients initially enrolled in the study, 639 were finally included and analyzed. The baseline characteristics of enrolled patients are summarized in Table 1. Among these patients, 109 (17.1%) received olanzapine (four‐antiemetic group) and 530 (82.9%) did not (three‐antiemetic group). The median age was 50 years (range, 27–80 years), and 64.2% of patients were < 55 years old. A total of 25.5%, 41.8%, and 54.6% of patients reported no alcohol consumption habit, a positive history of morning sickness, and being premenopausal, respectively. Four premenopausal patients (1.1%) received leuprorelin, a luteinizing hormone‐releasing hormone (LH‐RH) agonist, before the start of chemotherapy for ovarian protection. As for other anti‐endocrine drugs, no patient used them during the month before chemotherapy induction and during the observation period (first cycle) after chemotherapy induction.

**TABLE 1:** Baseline patient characteristics.

	Four‐antiemetic group (*n* = 109)		Three‐antiemetic group (*n* = 530)		All (*n* = 639)	
Characteristic	*n*	(%)	*n*	(%)	*n*	(%)
Age, years						
Median (range)	48 (32–72)		50 (27–80)		50 (27–80)	
<55 years	75	68.8	335	63.2	410	64.2
Gender						
Female	109	100	530	100	639	100
ECOG performance status						
0	108	99.1	521	98.3	629	98.4
≥1	1	0.9	9	1.7	10	1.6
BMI						
Median (range)	21.7 (17.0–32.0)		21.6 (14.7–39.6)		21.6 (14.7–39.4)	
<27.5 kg/m^2^	100	91.7	467	88.1	567	88.7
Alcohol consumption habits						
Yes	70	64.2	406	76.6	476	74.5
No	39	35.8	124	23.4	163	25.5
History of morning sickness						
Yes	49	45.0	218	41.1	267	41.8
No	60	55.0	312	58.9	372	58.2
Menopause^a^						
Yes (postmenopause)	41	37.6	249	47.0	290	45.4
No (premenopause)	68	62.4	281	53.0	349	54.6
Chemotherapy regimen						
AC	45	41.3	241	45.5	286	44.8
FEC100	64	58.7	289	54.5	353	55.2
Treatment setting						
Neoadjuvant	49	45.0	168	31.7	217	34.0
Adjuvant	60	55.0	362	68.3	422	66.0

Abbreviations: ECOG, Eastern Cooperative Oncology Group; BMI, body mass index; AC, doxorubicin 60 mg/m^2^ and cyclophosphamide 600 mg/m^2^ by intravenous drip infusion, all on Day 1, every 3 weeks; FEC100, fluorouracil 500 mg/m^2^, epirubicin 100 mg/m^2^, and cyclophosphamide 500 mg/m^2^ by intravenous drip infusion, all on Day 1, every 3 weeks.

^a^ Menopause was defined as amenorrhea lasting ≥12 months.

### 3.2 Incidence of CINV

A total of 9.5%, 37.1%, and 65.3% of patients experienced vomiting, ≥Grade 2 nausea, and CR failure, respectively. In separate analyses of the four‐antiemetic and three‐antiemetic groups, vomiting was reported by 5.5% and 10.4%, ≥Grade 2 nausea by 40.4% and 36.4%, and CR failure by 64.2% and 65.5% of patients, respectively.

### 3.3 Association between CINV and menopause: Risk factor analysis

Multicollinearity (VIF ≥10) between explanatory variables was not observed, so multivariate logistic regression analysis was performed using all planned explanatory variables. The results of univariate and multivariate logistic regression analyses are shown in Table 2. Factors showing a statistically significant association with vomiting in the multivariate analysis included no alcohol consumption habit (adjusted odds ratio [OR] = 2.264; 95% confidence interval [CI] = 1.257–4.079; *p* = 0.007), FEC100 regimen (adjusted OR = 2.585; 95% CI = 1.404–4.761; *p* = 0.002), and three‐antiemetic regimen without olanzapine (adjusted OR = 2.465; 95% CI = 1.010–6.020; *p* = 0.048). A history of morning sickness also showed a significant association with ≥Grade 2 nausea in the multivariate analysis (adjusted OR = 1.796; 95% CI = 1.290–2.500; *p* = 0.001). Factors that showed a significant association with CR failure in the multivariate analysis included a history of morning sickness (adjusted OR = 2.106; 95% CI = 1.486–2.986; *p* = 0.001) and FEC100 regimen (adjusted OR = 1.460; 95% CI = 1.041–2.048; *p* = 0.028). Premenopausal state was significantly associated with ≥Grade 2 nausea and CR failure in the univariate analysis but not in the multivariate analysis.

**TABLE 2:** Association between CINV and menopause.

	Univariate analysis		Multivariate analysis	
Factors	Crude OR (95%CI)	*p*‐value	Adjusted OR (95%CI)	*p*‐value
Vomiting				
Age (<55 years)	1.641 (0.905–2.976)	0.100	1.340 (0.534–3.365)	0.533
BMI (<27.5 kg/m^2^)	0.613 (0.296–1.268)	0.183	0.613 (0.289–1.300)	0.202
Alcohol consumption habits (No)	1.748 (1.003–3.049)	0.047*	2.264 (1.257–4.079)	0.007*
History of morning sickness^a^ (Yes)	1.497 (0.883–2.541)	0.132	1.443 (0.839–2.481)	0.185
Menopause^b^ (No)	1.418 (0.824–2.441)	0.205	1.347 (0.573–3.163)	0.494
Chemotherapy regimen (FEC100)	2.466 (1.362–4.463)	0.002*	2.585 (1.404–4.761)	0.002*
Antiemetic regimen (three‐antiemetic combination)	1.988 (0.833–4.741)	0.115	2.465 (1.010–6.020)	0.048*
≥Grade 2 nausea^c^				
Age (<55 years)	1.767 (1.249–2.500)	0.001*	1.514 (0.869–2.637)	0.143
BMI (<27.5 kg/m^2^)	0.859 (0.521–1.418)	0.552	0.810 (0.485–1.354)	0.422
Alcohol consumption habits (No)	1.214 (0.843–1.747)	0.298	1.324 (0.903–1.942)	0.151
History of morning sickness^a^ (Yes)	1.829 (1.320–2.534)	0.001*	1.796 (1.290–2.500)	0.001*
Menopause^b^ (No)	1.617 (1.166–2.243)	0.004*	1.234 (0.728–2.093)	0.434
Chemotherapy regimen (FEC100)	1.391 (1.004–1.926)	0.047*	1.323 (0.945–1.851)	0.103
Antiemetic regimen (three‐antiemetic combination)	0.846 (0.555–1.290)	0.437	0.930 (0.601–1.439)	0.745
CR failure				
Age (<55 years)	1.534 (1.096–2.147)	0.012*	1.243 (0.717–2.156)	0.438
BMI (<27.5 kg/m^2^)	0.932 (0.554–1.567)	0.790	0.899 (0.527–1.534)	0.697
Alcohol consumption habits (No)	0.918 (0.633–1.331)	0.651	0.989 (0.670–1.461)	0.957
History of morning sickness^a^ (Yes)	2.132 (1.510–3.011)	0.001*	2.106 (1.486–2.986)	0.001*
Menopause^b^ (No)	1.486 (1.071–2.062)	0.017*	1.260 (0.735–2.159)	0.401
Chemotherapy regimen (FEC100)	1.546 (1.114–2.146)	0.009*	1.460 (1.041–2.048)	0.028*
Antiemetic regimen (three‐antiemetic combination)	1.056 (0.687–1.625)	0.803	1.142 (0.731–1.784)	0.561

Abbreviations: CINV, chemotherapy‐induced nausea and vomiting; BMI, body mass index; FEC100, fluorouracil 500 mg/m^2^, epirubicin 100 mg/m^2^, and cyclophosphamide 500 mg/m^2^ by intravenous drip infusion, all on Day 1, every 3 weeks; OR, odds ratio; CI, confidence interval; CR, complete response.

^a^ Patients who had never been pregnant were counted as having no history of morning sickness.

^b^ Menopause was defined as amenorrhea lasting ≥12 months.

^c^ Nausea was graded according to Common Terminology Criteria for Adverse Events, version 5.0.

* Statistically significant.

### 3.4 Association between CINV and FSH/E2

FSH and E2 levels were measured before the start of treatment in 137 patients (premenopausal FSH/E2 group: 121 patients; postmenopausal FSH/E2 group: 16 patients). The mean (range) in FSH level was 18.3 mIU/mL (2.0–131.1) in the premenopausal FSH/E2 group and 84.1 mIU/mL (48.1–118.6) in the postmenopausal FSH/E2 group. E2 level was 109.7 pg/mL (14.0–797.1) and 15.8 pg/mL (14.0–19.0), respectively.

In the premenopausal and postmenopausal FSH/E2 groups, there was no significant difference between the two groups in terms of the percentage of patients on the FEC100 regimen who reported no alcohol consumption habit (22.3% vs. 25.0% and 55.4% vs. 56.3%, respectively). The percentage of patients with a history of morning sickness was higher in the premenopausal FSH/E2 group (48.8% vs. 31.3%), and the percentage of patients on the three‐antiemetic regimen without olanzapine was higher in the postmenopausal FSH/E2 group than premenopausal FSH/E2 group (87.6% vs. 100%, respectively).

**FIGURE 2:**
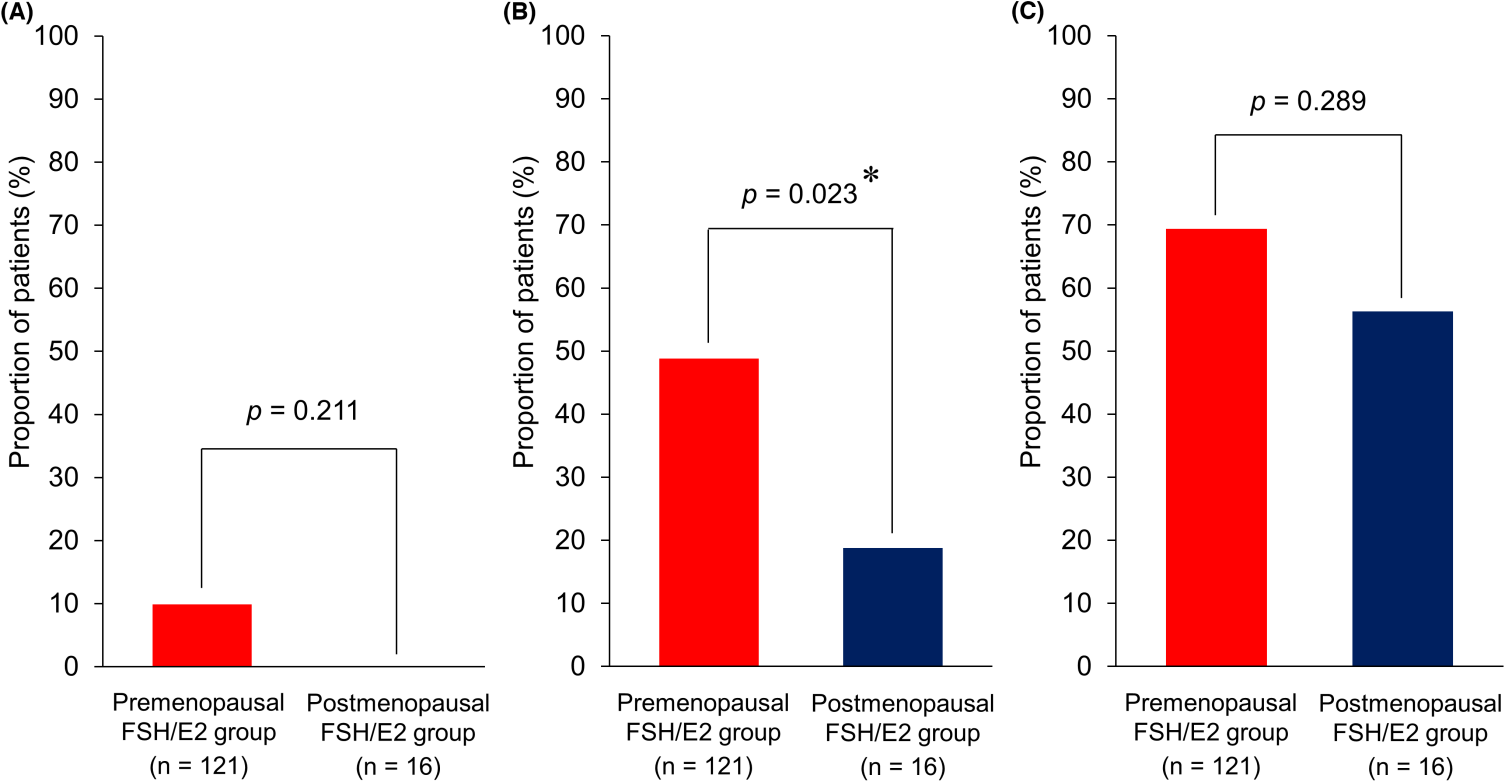
Associations between CINV and FSH/E2.(A) Vomiting, (B) ≥Grade 2 nausea, and (C) Complete response failure. CINV rates were compared between the premenopausal FSH/E2 group (i.e., FSH <40 mIU/mL and E2 ≥20 pg/mL) and the postmenopausal FSH/E2 group (i.e., FSH ≥40 mIU/mL and E2 <20 pg/mL) using the chi‐square test or Fisher's exact test. The premenopausal FSH/E2 group had significantly higher ≥Grade 2 nausea than the postmenopausal FSH/E2 group (48.8% vs 18.8%, *p* = 0.023). FSH, follicle‐stimulating hormone; E2, estradiol. *Statistically significant.

An analysis of the association between CINV and FSH/E2 revealed that the premenopausal FSH/E2 group had a significantly higher incidence of ≥Grade 2 nausea than the postmenopausal FSH/E2 group (48.8% vs. 18.8%, *p* = 0.023). Although the proportion of patients experiencing vomiting and CR failure tended to be higher in the premenopausal FSH/E2 group, the difference was not significant in either case (Figure 2).

In a separate analysis of FSH and E2, the low FSH group had a significantly higher incidence of ≥Grade 2 nausea than the high FSH group (50.9% vs. 25.8%, *p* = 0.013). There was also a trend toward a higher incidence of ≥Grade 2 nausea in the high E2 group compared to the low E2 group (48.3% vs. 28.6%, *p* = 0.095), although the difference was not significant (Table 3).

**TABLE 3:** Associations between CINV and FSH as well as CINV and E2.

	FSH level			E2 level		
	Low (*n* = 106)	High (*n* = 31)	*p*‐value	Low (*n* = 21)	High (*n* = 116)	*p*‐value
Vomiting (%)	10.4	3.2	0.195	14.3	7.8	0.271
≥Grade 2 nausea^a^ (%)	50.9	25.8	0.013*	28.6	48.3	0.095
CR failure (%)	70.8	58.1	0.183	66.7	68.1	0.897

Abbreviations: CR, complete response; E2, estradiol; FSH, follicle‐stimulating hormone.

^a^ Nausea was graded according to Common Terminology Criteria for Adverse Events, version 5.0.

* Statistically significant.

## 4 DISCUSSION

This study clarified the relationship of menopause and FSH/E2 with CINV in HEC for early‐stage breast cancer using a sample size sufficient for validation. The multivariate analysis showed that premenopausal state, defined as a physician's diagnosis of amenorrhea lasting <12 months, was not a significant risk factor for CINV, whereas a premenopausal state defined based on serum levels of FSH and E2 (i.e., FSH <40 mIU/mL and E2 ≥20 pg/mL) was associated with a significantly increased incidence of ≥Grade 2 nausea. Also, in separate analyses of FSH and E2, the incidence of ≥Grade 2 nausea was significantly higher in the low FSH group (FSH <40 mIU/mL) and tended to be higher in the high E2 group (E2 ≥20 pg/mL), indicating the above mentioned premenopausal FSH/E2 levels (FSH <40 mIU/mL and E2 ≥20 pg/mL). This is a novel finding not reported in previous studies. Furthermore, a history of morning sickness was significantly associated with both ≥Grade 2 nausea and CR failure, in agreement with previous studies.^12^, ^13^

Previous studies reported that pregnant women with elevated levels of hCG and E2 (a type of estrogen) have more‐severe morning sickness symptoms.^25^, ^26^ The present study found associations between CINV and serum levels of FSH and E2 (i.e., FSH <40 mIU/mL and E2 ≥ 20 pg/mL) or a history of morning sickness. In addition, the incidence of ≥Grade 2 nausea was significantly lower in the postmenopausal FSH/E2 group than the premenopausal FSH/E2 group, and none of these patients received olanzapine and there were no cases of vomiting. The findings of this study thus suggest that increased estrogen secretion may exacerbate CINV.

Although the mechanism by which increased estrogen secretion exacerbates CINV is unknown, one study reported a strong association between single‐nucleotide polymorphisms in estrogen‐responsive sequences of the *TACR1* gene, which encodes the NK_1_ receptor, and development of PONV.^27^ Higher *TACR1* methylation rates were shown to be associated with lower PONV incidence in women.^27^ A study of anthracycline‐based chemotherapy for breast cancer revealed an association between the *TACR1* gene and the delayed phase of CINV.^28^ One mechanism of CINV involves the binding of substance P to the NK_1_ receptor, and a significant increase in plasma substance P levels has been reported, especially in the delayed phase of CINV.^29^ Palonosetron, a 5‐HT_3_ RA, specifically inhibits “crosstalk” between the NK_1_ and 5‐HT_3_ receptor signaling pathways^30^, ^31^, ^32^ and is reportedly more effective against delayed‐phase CINV than conventional 5‐HT_3_ RAs.^33^, ^34^, ^35^ Furthermore, a subgroup analysis of the PROTECT study^33^ reported that palonosetron is particularly effective in patients <55 years old and women. Studies in rats examining the gene and protein expression of neuropeptides and their receptors, including substance P and TACR1, revealed decreased levels of serum estrogen and TACR1 in the brain in the bilateral ovariectomized group compared with controls.^36^ These reports suggest that female hormones such as estrogen are involved in the regulation of *TACR1* expression and can thereby exacerbate CINV.

This study has two main limitations. First, there were limitations in setting the observation period. Since experience with CINV has been reported to increase the risk of developing CINV,^37^ the first cycle without this effect was used as the observation period for this study. However, because this was a retrospective study, it was not possible to conduct a detailed analysis of the acute and delayed phases of CINV. The mechanism of CINV differs between the acute and delayed phases, with the acute phase primarily affected by serotonin and the delayed phases by substance P.^29^, ^32^, ^38^, ^39^ In addition, the generalizability of the study results is limited because data from only a single center were examined in this study. Therefore, in future multicenter prospective studies, it will be necessary to analyze both phases separately by setting an appropriate observation period, such as 120 h after the start of chemotherapy. Second, FSH and E2 were measured in only 21.4% of the patients (137/639), and we were unable to measure serum FSH and E2 levels after the start of chemotherapy. Also, serum concentrations of female hormones other than FSH and E2 (e.g., progesterone) were not measured and could not be included in the analysis. In premenopausal women, these values fluctuate according to menstrual status. In addition, menopausal transition (perimenopause) is prone to changes in FSH and E2 levels.^40^ Therefore, not only the criterion of “amenorrhea lasting more than 12 months” but also the menopausal criterion in serum FSH and E2, etc. levels prior to chemotherapy should be considered. In the present study, approached from these two perspectives, we found that pre‐chemotherapy serum FSH and E2 levels are potential indicators of the risk of developing severe nausea. While this is a major step forward, further investigation is needed in light of the limitations of the aforementioned studies. As an additional future study, further analyses of changes in blood levels of FSH/E2 and substance P and the effect of *TACR1* expression should provide a more‐detailed understanding of the mechanism of CINV exacerbation and thereby aid in the realization of individualized antiemetic therapies.

In conclusion, our results suggest that a premenopausal state affects the severity of CINV and that levels of FSH and E2 (especially FSH) may be useful indicators for CINV risk assessment.

## AUTHOR CONTRIBUTIONS

**Takashi Yokokawa:** Conceptualization (lead); investigation (lead); methodology (equal); project administration (lead); supervision (equal); visualization (lead); writing – original draft (lead); writing – review and editing (lead). **Kenichi Suzuki:** Conceptualization (supporting); methodology (equal); project administration (supporting); writing – review and editing (supporting). **Daiki Tsuji:** Conceptualization (supporting); methodology (equal); project administration (supporting); writing – review and editing (supporting). **mari hosonaga:** Conceptualization (supporting); methodology (supporting); project administration (supporting); writing – review and editing (supporting). **Kazuo Kobayashi:** Data curation (lead); formal analysis (lead); methodology (supporting); writing – review and editing (supporting). **Kazuyoshi Kawakami:** Investigation (equal); methodology (supporting); project administration (supporting); writing – review and editing (supporting). **Hitoshi Kawazoe:** Methodology (supporting); writing – review and editing (supporting). **Tomonori Nakamura:** Methodology (supporting); writing – review and editing (supporting). **Wataru Suzuki:** Investigation (supporting); writing – review and editing (supporting). **Takahito Sugisaki:** Investigation (supporting); writing – review and editing (supporting). **Takeshi Aoyama:** Investigation (supporting); writing – review and editing (supporting). **Koki Hashimoto:** Investigation (supporting); writing – review and editing (supporting). **Masahiro Hatori:** Investigation (supporting); writing – review and editing (supporting). **Takuya Tomomatsu:** Investigation (supporting); writing – review and editing (supporting). **Ayaka Inoue:** Investigation (supporting); writing – review and editing (supporting). **Keiichi Azuma:** Investigation (supporting); writing – review and editing (supporting). **Maimi Asano:** Investigation (supporting); writing – review and editing (supporting). **Toshimi Takano:** Writing – review and editing (supporting). **Shinji Ohno:** Writing – review and editing (supporting). **Masakazu Yamaguchi:** Project administration (supporting); supervision (equal); writing – review and editing (supporting).

## FUNDING INFORMATION

This study was supported by a research grant from the Japanese Society of Pharmaceutical Oncology.

## CONFLICT OF INTEREST STATEMENT

Hitoshi Kawazoe received research funding from Eli Lilly. Tomonori Nakamura received research funding from Astellas Pharma, Chugai, Daiichi Sankyo, Kyowa Kirin, Otsuka Pharmaceutical, Sanofi, Sato Pharmaceutical, and Shionogi. Toshimi Takano received honoraria for lectures from Celltrion Healthcare, Chugai, Daiichi‐Sankyo, Eisai, and Eli Lilly. Shinji Ohno received honoraria for lectures from AstraZeneca, Chugai, Eisai, Eli Lilly, and Pfizer. The remaining authors declare that the research was conducted in the absence of any commercial or financial relationships that could be construed as a potential conflict of interest.

## ETHICS STATEMENT

The study protocol was approved by the Clinical Research Ethics Review Committee of the Cancer Institute Hospital (approval number: 2019‐GA‐1238) and conducted in accordance with the Declaration of Helsinki.

## PATIENT CONSENT STATEMENT

The ethics committee waived the need for consent due to the retrospective nature of the study.

## Data Availability

The data that support the findings of this study are available from the corresponding author upon reasonable request. The data are not publicly available due to privacy or ethical restrictions.
